# Case series of three malaria patients from Thailand infected with the simian parasite, *Plasmodium cynomolgi*

**DOI:** 10.1186/s12936-022-04167-w

**Published:** 2022-05-06

**Authors:** Piyaporn Sai-ngam, Kingkan Pidtana, Preeyaporn Suida, Kamonporn Poramathikul, Paphavee Lertsethtakarn, Worachet Kuntawunginn, Sarayut Tadsaichol, Montri Arsanok, Siriporn Sornsakrin, Chaiyaporn Chaisatit, Chaiyawat Mathavarat, Sasikanya Thaloengsok, Parat Boonyarangka, Chadin Thongpiam, Samandra Demons, Brian Vesely, Norman C. Waters, Aungkana Saejeng, Mariusz Wojnarski, Sutchana Tabprasit, Chokchai Kwanpichit, John S. Griesenbeck, Michele Spring

**Affiliations:** 1 US Army Armed Forces Research Institute of Medical Sciences, Bangkok, Thailand; 2 Ministry of Public Health (MoPH), Vector Borne Disease Control Center 12.1, Yala, Thailand; 3 Southern Border Provinces Medical Center, Yala, Thailand; 4 US Army Medical Materiel Development Activity, Fort Detrick, MD USA; 5 Ministry of Public Health, Division of Vector Borne Diseases, Nonthaburi, Thailand; 6 Royal Thai Army-Army Armed Forces Research Institute of Medical Sciences, Bangkok, Thailand; 7 Royal Thai Army-Forward Internal Security Operation Command Region 4, Yala, Thailand; 8 The Henry M. Jackson Foundation for the Advancement of Military Medicine, Inc, Bethesda, MD USA

**Keywords:** Malaria, Simian, Thailand, Plasmodium, Cynomolgi, Macaques, Human

## Abstract

**Background:**

While human cases of *Plasmodium knowlesi* are now regularly recognized in Southeast Asia, infections with other simian malaria species, such as *Plasmodium cynomolgi*, are still rare. There has been a handful of clinical cases described, all from Malaysia, and retrospective studies of archived blood samples in Thailand and Cambodia have discovered the presence *P. cynomolgi* in isolates using polymerase chain reaction (PCR) assays.

**Case presentation:**

In Thailand, an ongoing malaria surveillance study enrolled two patients from Yala Province diagnosed with *Plasmodium vivax* by blood smear, but who were subsequently found to be negative by PCR. Expanded PCR testing of these isolates detected mono-infection with *P. cynomolgi*, the first time this has been reported in Thailand. Upon re-testing of 60 isolates collected from Yala, one other case was identified, a co-infection of *P. cynomolgi* and *P. vivax*. The clinical course for all three was relatively mild, with symptoms commonly seen in malaria: fever, chills and headaches. All infections were cured with a course of chloroquine and primaquine.

**Conclusion:**

In malaria-endemic areas with macaque populations, cases of simian malaria in humans are being reported at an increasing rate, although still comprise a very small percentage of total cases. *Plasmodium cynomolgi* and *P. vivax* are challenging to distinguish by blood smear; therefore, PCR can be employed when infections are suspected or as part of systematic malaria surveillance. As Thai MoPH policy schedules regular follow-up visits after each malaria infection, identifying those with *P. cynomolgi* will allow for monitoring of treatment efficacy, although at this time *P. cynomolgi* appears to have an uncomplicated clinical course and good response to commonly used anti-malarials.

## Background

The first naturally-acquired human infection of the simian malaria parasite, *Plasmodium cynomolgi*, was reported from Malaysia in 2014 [1]. Clinical cases have continued to be reported from Malaysia, and *P. cynomolgi* has been retrospectively detected in stored isolates from Malaysia, Cambodia and Thailand [2–8]. An ongoing malaria surveillance study in Thailand has been enrolling malaria patients to monitor transmission in border provinces and determine resistance patterns in order to better manage and predict effectiveness of anti-malarial treatments. As malaria cases continue to decrease in Thailand, it will become important for such surveillance studies to more actively monitor for human infections by simian malaria parasites.

## Malaria case presentations

**Fig. 1:**
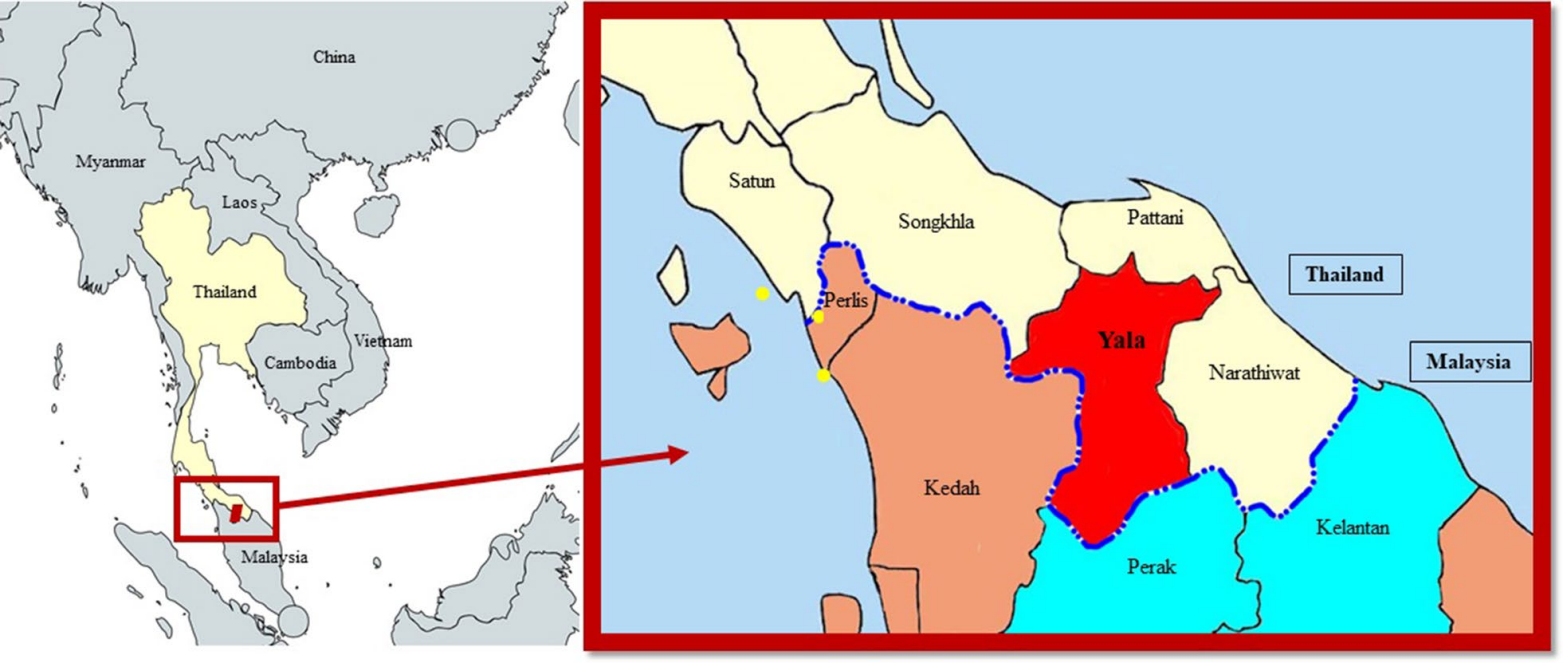
Location of human *P. cynomolgi* cases in Thailand. Map of Yala Province, Thailand with location of detected human *P. cynomolgi* cases (yellow dots). The royal blue dotted line indicates the border between Thailand and Malaysia. Provinces in light yellow and red are located in Thailand, and those that are brown and light blue are in Malaysia, with the two states of Perak and Kelantan being two areas with previously reported human *P. cynomolgi* cases [7]

This minimal risk malaria surveillance study in Thailand has been enrolling individuals diagnosed with malaria by rapid diagnostic test (RDT) and/or microscopy since March 2019. The study operates in several border provinces: Yala (by Malaysia), Sisaket and Ubon Ratchathani (by Cambodia), and Ratchaburi (by Myanmar). After consent, a single venous blood sample is drawn, with a complete blood count (CBC), glucose 6-phosphate dehydrogenase (G6PD) CareStart™ RDT (Access Bio, Inc., USA) and fluorescent spot testing (R&D Diagnostics Ltd., Greece) performed by local Ministry of Public Health (MoPH) or Royal Thai Army (RTA) staff. The remaining blood sample shipped to US Armed Forces Research Institute of Medical Sciences (AFRIMS) in Bangkok, Thailand. There, speciation is verified by blood smears that are made and read by AFRIMS staff, and by conducting multiplex real time polymerase chain reaction (RT-PCR) on isolated parasite DNA. In addition, quantitative G6PD testing (Pointe Scientific, USA), PCR for molecular markers of resistance and submicroscopic gametocytaemia as well as *ex-vivo* and *in-vitro* drug susceptibility assays are performed. At the time of writing, 149 malaria patients have been enrolled: 128 *Plasmodium vivax* cases, 14 *Plasmodium falciparum* and four *Plasmodium knowlesi* cases*.* Three infections with *P. cynomolgi* were also detected. A short description of these, and the locations within Yala Province, Thailand (Fig. 1), follows.

### Case A

A 53-year-old woman presented at a malaria clinic in Ban Nang Sata District, in March 2021 with 38 °C fever, headache, and chills for five days. The haematological assessment showed white blood count (WBC) at 4,200/mm^3^, haemoglobin at 10.9 g/dL, and platelets at 191,000/mm^3^. She reported working at a rubber plantation, and that her husband had recently been diagnosed and treated for *P. vivax* infection.

### Case B

A 55-year-old female rubber plantation worker was part of a malaria active case detection investigation by malaria clinic staff from Ka Bang District, in February 2021. The patient reported a history of headache and fever for eight days, although on the day of examination, the subject’s tympanic temperature was 37 °C. Laboratory examination revealed WBC at 4800/mm^3^, haemoglobin at 11.7 g/dL, and platelet count at 330,000/mm^3^.

### Case C

In June 2021, a 25-year-old male on active duty in the Royal Thai Army presented at a malaria clinic in Yala District, with a complaint of five days of fever and nighttime chills. His temperature was 37.8 °C. Haematology findings showed slight thrombocytopenia at 123,000/mm^3^, WBC at 6900/mm^3^, and haemoglobin at 12.5 g/dL. The patient stated he had been stationed in Yala District for at least 20 months, going out on daily patrols and sleeping overnight in the forest. He reported using mosquito repellent and mosquito coils for personal protection.

Using microscopy, all three subjects were diagnosed with *P. vivax*; all presented with uncomplicated illness, had normal G6PD activity and reported no prior history of malaria. Each patient was treated by local health care staff with three days of chloroquine and a 2-week radical cure course of primaquine, as per Thai national treatment guidelines. All were found to be clinically well within 5 days of initiating the anti-malarials, with no recurrences at subsequent follow-up visits required by the Thai MoPH scheduled at 14-, 28-, 60- and 90-days post-treatment.

## Laboratory investigations

**Fig. 2:**
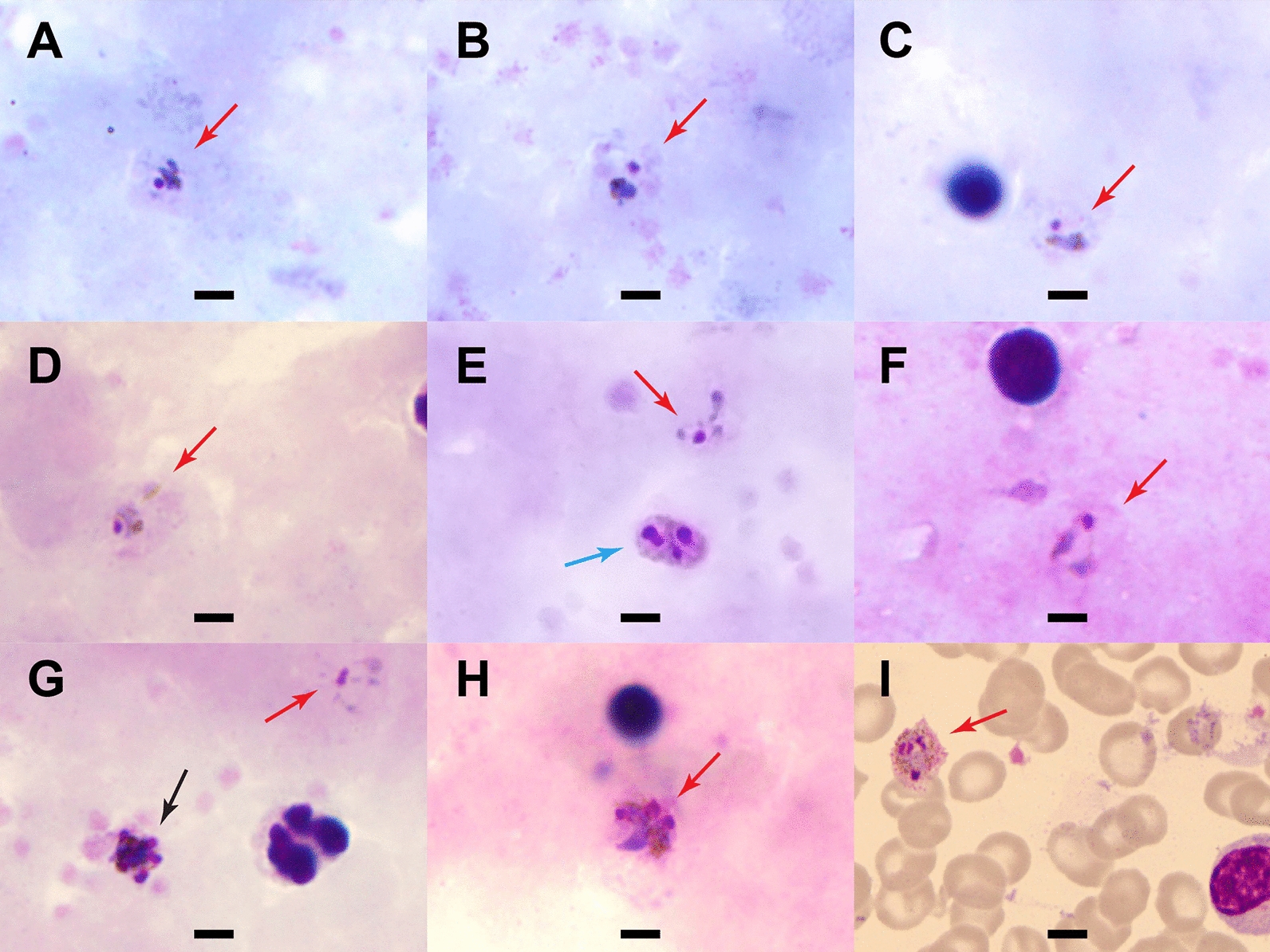
Parasite morphology in Giemsa-stained blood smears from the three malaria patients. Shown are malaria parasites detected in Giemsa-stained films at a magnification of 100x. **A**-**E** Case A (thick film) showing growing trophozoite stages with amoeboid-shaped cytoplasm (red arrows). Yellowish-brown pigments were visible (**A**-**D**) with double chromatin dots in E (blue arrow). **F** Case B (thick film) with growing trophozoite stages. **G** and **H** Case C (thick film). Parasites resembling *P. vivax* were found in the field of view (**G**, red arrow). Early schizont with merozoites was also seen in **G** (black arrow) and triple chromatin dots in **H**. **I** Case C (thin film) with dominant Schüffner’s stippling (pink, scattered dots) and yellowish-brown pigments in a trophozoite. Erythrocytes did not appear enlarged. Scale bar indicates 5 µm

Blood smears were prepared and read by two World Health Organization (WHO)-certified microscopists at the AFRIMS labs in Bangkok, Thailand. In brief, thick and thin smears were prepared on the same glass slide and air-dried and fixed in methanol, stained for 45 min (min) in 3% diluted Giemsa stain, and examined at an oil immersion magnification of × 100. Parasite counting was done per 500 white blood cells (WBC) in thick films, and percent parasitaemia was calculated based on the actual WBC count. Parasites resembling *P. vivax* were detected, with densities of 25, 10, and 2718 parasites/µL blood for Case A, B, and C, respectively. Only Case C had gametocytaemia, with four gametocytes per 200 WBCs, or 138 gametocytes/µL. Malaria parasite morphologies in Giemsa-stained thick blood smears are shown in Fig. 2A–H, demonstrating growing trophozoite stages with amoeboid-shaped cytoplasm (red arrows). No ring forms were detected in any slide. Single (Panels A–D, F), double (blue arrow, Panel E), and triple (Panel H) chromatin dots were seen on examination. There was yellowish-brown pigment dispersed within the cytoplasm in some infected cells. In thin films, parasites were found only in Case C (Fig. 2I), the individual with mixed infection and higher parasite count. The erythrocytes were not clearly enlarged or distorted, and Schüffner's stippling was prominently visible.

The PCR testing performed at AFRIMS is designed to detect five *Plasmodium* species: *P. falciparum*, *P. vivax*, *Plasmodium malariae*, *Plasmodium ovale* and *P. knowlesi*. Briefly, parasite genomic DNA is extracted from whole blood collected in ethylenediaminetetraacetic acid (EDTA) using EZ1 DNA blood kit with automated EZ1 Advanced XL purification system (QIAGEN, Valencia, CA, USA), and *Plasmodium* speciation confirmed by multiplex RT- PCR, using species-specific primers and probes [9, 10]. Two of the study patients (A and B) were found to be negative by multiplex RT-PCR, with *P. vivax* reported for Case C.

Since asexual parasites had been observed on blood smear for Cases A and B, further investigations were undertaken to identify the species. The 5-species multiplex RT- PCR was re-run as well as a singleplex RT-PCR testing for *P. cynomolgi.* Primers and probes specific to small subunit rRNA, S-type (Genbank accession number L08242.1 were selected, with sequences as follows: Forward: 5′-ATTGCGGTCGCAAATAATGAAG-3′, Reverse: 5′-GGTATGATAAGCCAGGGAAGTG-3′ and Probe: 5′-FAM-TACTCGCTCCTTCTGTTCCCTGGA-BHQ1′). The reaction was carried out in a 25 µl reaction using Rotor-Gene Multiplex PCR kit (QIAGEN, Hilden, Germany) with cycling conditions consisting of an initial activation step at 95 °C for 5 min, followed by 45 cycles of denaturation at 95 °C for 15 s and annealing /extension at 60 °C for 15 s. Blood from a macaque infected with *P. cynomolgi* was used as a positive control. Mono-infection with *P. cynomolgi* was confirmed by PCR in Cases A and B, with Case C having co-infection with *P. vivax*. All remaining Yala samples (n = 60) were then tested for *P. cynomolgi* by singleplex RT-PCR and were negative.

## Discussion

*Plasmodium cynomolgi* is a malaria species with Southeast Asian macaques as a natural host, transmitted through the bites of the forest-dwelling, Leucosphyrus Group of *Anopheles* mosquitoes, which exhibits relapses upon activation of hypnozoites similar to *P. vivax* [4, 7, 11, 12]. This report describes three individuals enrolled in a malaria surveillance study in Thailand who were found to have *P. cynomolgi* infection, although after an initial microscopic diagnosis of and treatment for *P. vivax*. The morphologic characteristics shown on the blood films in Fig. 2 are present in both species, with similarities also evident at the structural level as described by Kosaisavee et al. [13]. For Case C, who harboured co-infection with *P. cynomolgi* and *P. vivax*, it was not possible to identify individual parasite species accurately, even in the thin film, and the parasitaemias in Cases A and B were too low to confidently locate parasites and characterize morphology. Malaria RDTs currently in use are not adequate diagnostic tools for *P. cynomolgi*. Test antigens are either pan-*Plasmodium* (e.g., aldolase or lactate dehydrogenase (LDH)) or *P. falciparum* or *P. vivax* specific, and the sensitivity in pan-*Plasmodium* RDTs detecting non-falciparum or non-vivax species of malaria is quite variable [14]. Cross- reactivity between *P. vivax* and *P. cynomolgi* LDH in laboratory setting has recently been demonstrated [15], but it is not clear this would translate to accuracy in a field-deployed RDT. In addition, the low parasitaemias seen in *P. cynomolgi* may further reduce RDT sensitivity. With the difficulties in diagnosis by blood smear even for qualified/experienced microscopists, and the lack of utility for RDTs, diagnostic testing by PCR or other molecular methods is likely to be required.

The only other publication on *P. cynomolgi* prevalence in Thailand conducted PCR assays on 1152 archived samples from malaria patients in Tak, Ubon Ratchathani, Chanthaburi, Yala, and Narathiwat Provinces during the period of 2007 to 2017 [8]. There were nine *P. cynomolgi* infections detected, all co-infections: *P. cynomolgi* with *P. vivax* (n = 7), with *P. falciparum* (n = 1), or with both *P. vivax* and *P. knowlesi* (n = 1). Cases were distributed across various years, diagnosed between April and December (rainy season is May–October), and found in all provinces, although Yala had five of the nine cases (55%). In these *P. cynomolgi* clinical cases from 2021, two of the three were mono-infections, which is the first time this has been reported in Thailand. There is one case report of *P. cynomolgi* mono-infection from a European tourist traveling through Thailand (Surat Thani Province) and Malaysia [3]. However, the origin of infection could not be confirmed.

With an initial microscopic diagnosis of *P. vivax*, the patients were not questioned for a history of contact with macaques. At the follow-up visits by the Yala study team, Case A and B did report the presence of macaques near their homes. In Thailand, the main hosts of *P. cynomolgi*, *P. knowlesi*, *Plasmodium inui*, and *Plasmodium coatneyi* are *Macaca fascicularis* and *Macaca nemestrina*, with recent reports in stump-tailed macaques, *Macaca arctoides* [16]. Co-infections of simian malaria are not uncommon in macaques, with the presence of two or three species simultaneously detected in 18% to 40% of monkeys [16, 17], which may explain why some human studies report co-infections more than mono-infections [2, 5]. *Plasmodium cynomolgi* was first reported as a mono-infection in a Malaysian woman in 2014 [1], and up to now, cases have been shown to exist in both peninsular Malaysia and Borneo Malaysia, the latter where *P. knowlesi*, another simian malaria is endemic [5, 7]. There have been six other studies reporting the prevalence of *P. cynomolgi* in humans in Southeast Asia, shown in Table 1.

**Table 1:** Summary of literature on *P. cynomolgi* cases in Southeast Asia

Location	Sample set	N	Diagnosis by PCR
Borneo Malaysia (Sarawak)^2^	Malaria patients	332	All mixed infections: *P. cynomolgi* and *P. knowlesi* (n = 5)
Borneo Malaysia (Sabah)^4^	Survey for asymptomatic, low-density malaria cases	876	*P. cynomolgi* (n = 2)
Borneo Malaysia (Kapit)^5^	Malaria patients	1,047	All mixed infections: *P. cynomolgi* & *P. knowlesi* (n = 6)
Peninsular Malaysia^7^	Survey of communities living at forest fringe	645	*P. cynomolgi* (n = 9)
Cambodia (Pailin/Battambang)^6^	Survey of asymptomatic submicroscopic malaria cases	1361	*P. cynomolgi* (n = 11) Mixed infection of *P. cynomolgi* & *P. vivax* (n = 2)

Data on prevalence of *P. cynomolgi* taken from references 2, 4–7 is summarized. Columns from left to right: location names country and province/state of the study, samples set describes from which population blood samples were collected, and N is number of samples tested by PCR. Diagnosis by PCR presents number of isolates found to have *P. cynomolgi* mono-infections or *P. cynomolgi* mixed infections

To date, most of the publications reporting on human *P. cynomolgi* infections are retrospective testing of blood samples. In the two clinical case reports of mono-infection, and past experimental infections in humans [1, 2, 18], undifferentiated flu-like symptoms have been present, with symptoms occurring at very low parasitaemias and not progressing in severity. In humans, the anti-malarial treatment required for *P. cynomolgi* is not well studied, but macaques in *P. cynomolgi* drug and vaccine studies respond well to chloroquine and primaquine, the regimen for *P. vivax* in Thailand [19]*.* All the patients from Yala recovered rapidly, and there were no recurrences over three months of active follow-up. The low prevalence of simian malarias infecting humans means the parasites are not under frequent anti-malarial drug selection pressure and should remain susceptible to treatment [6]. In the study by Imwong et al. [6], two Cambodian individuals were found to have *P. cynomolgi* again three months after the initial diagnosis, but it was not possible to conclude whether it was a relapse, new infection, or persistent blood-stage infection.

The *P. cynomolgi* survey by Putaporntip et al. [8] demonstrated that *P. cynomolgi* has been infecting humans in Thailand for the last 15 years and is likely underdiagnosed. However among the published studies reviewed here, the prevalence of *P. cynomolgi* has been less than 1.5% in samples tested. In Thailand, the first clinical case *P. knowlesi* was reported in 2004, and by 2017, cases began to be regularly reported by the Thailand MoPH, peaking at 53 cases in 2021 [20, 21]. It is not yet understood if the increases in human simian malaria infections are due to better detection methods, the result of human encroachment into macaque habitats, or both. The three Yala patients were diagnosed separately in time and space, although Yala province borders with Perak and Kelantan States in Malaysia where *P. cynomolgi* has been documented [7, Fig. 1]. Whole-genome sequencing of the isolates is planned, which will allow lineage comparisons among these three cases as well as with data available from cases in the neighboring Malaysian states [7]. To mitigate the potential spread of *P. cynomolgi* and *P. knowlesi* and remain on track for malaria elimination, increased vigilance will be required for any signs of increased transmission in Yala and other areas in Thailand where exposure to macaques is possible.

## Conclusions

This cases series is the second time human *P. cynomolgi* infections have been documented in Thailand and the first report of mono-infections, along with a description of the clinical course of each. *P. cynomolgi* is quite challenging to distinguish from *P. vivax* microscopically, and while this may lead to underdiagnosis, the disease course is usually mild and should be adequately and rapidly treated using antimalarial regimens for *P. vivax*. Molecular characterization is the most accurate way to detect these rare infections, but the capabilities may not reach the areas that need it most. Going forward, for all samples collected during this malaria surveillance study, primers for *P. cynomolgi* will be included for 6-species real time PCR verification. Although the diagnoses may not be available before treatment is administered, the results will allow for a more accurate estimation of infection prevalence in Thailand and evaluation of treatment efficacy during the 90-day Thai MoPH follow-up period.

## Data Availability

The majority of the data generated is presented in this article, but requests may be made to the corresponding author. Permission from Thai MoPH and Royal Thai Army will also be required.

## Abbreviations

AFRIMS: Armed Forces Research Institute of Medical Sciences
DNA: Deoxyribonucleic acid
EDTA: Ethylenediaminetetraacetic acid
G6PD: Glucose 6-phosphate dehydrogenase
MoPH: Ministry of Public Health
PCR: Polymerase chain reaction
rRNA: Ribosomal ribonucleic acid
RTA: Royal Thai Army
WBC: White blood cell
WHO: World Health Organization
